# Learning curve for flexible bronchoscope-guided orotracheal intubation for anesthesiology residents: A cumulative sum analysis

**DOI:** 10.1371/journal.pone.0288617

**Published:** 2023-07-13

**Authors:** Xingzhi Cai, Mingming Yue, Xiaohui Liu, Lize Zhang, Shanshan Wu, Wenlong Shen, Ailan Yu

**Affiliations:** 1 Department of Anesthesiology, Liaocheng People’s Hospital, Liaocheng, Shandong, China; 2 Shanddong First Medical University, Shandong, China; Medical School, University of Pecs, HUNGARY

## Abstract

**Background:**

Endotracheal intubation with a flexible bronchoscope is a well-recognized airway management technique that anesthesiologists must master. Skill acquisition and knowledge must reach an appropriate level before trainees perform independent practice on patients. There are a paucity of evidence-based outcome measures of trainee competence in performing flexible bronchoscopy. The objectives of this study were to 1) construct a learning curve for flexible bronchoscope-guided orotracheal intubation for anesthesiology residents using the CUSUM method and 2) determine the number of procedures required to achieve proficiency.

**Methods:**

This study included 12 first-year anesthesiology residents with no previous experience with flexible bronchoscopic intubation. Trainees attended theoretical and simulation training and performed flexible bronchoscope-guided orotracheal intubation in adult patients with normal airways under general anesthesia. Number of intubation attempts, intubation success rate, time to intubation, and incidence of dental and mucosal injuries were recorded. The cumulative sum (CUSUM) method was used to evaluate the learning curve of flexible bronchoscope-guided orotracheal intubation.

**Results:**

Trainees performed flexible bronchoscope-guided orotracheal intubation on 364 patients. First-attempt intubation success occurred in 317 (87.1%) patients. Second-attempt intubation success occurred in 23 (6.3%) patients. Overall, the flexible bronchoscope-guided orotracheal intubation success rate was 93.4% (range, 85.3% to 100%). The mean number of orotracheal intubation procedures per trainee was 31 ± 5 (range, 23 to 40). All trainees crossed the lower decision boundary (H_0_) after 15.1 ± 5.6 procedures (range, 8 to 25 procedures). There was a significant decrease in median intubation time [39s (IQR: 30, 50) vs. 76s (IQR: 54, 119)] (*P* < 0.001) after crossing the lower decision boundary (H_0_) compared to before. There were no dental, mucosa, arytenoid or vocal cord trauma events associated with intubation.

**Conclusions:**

Learning curves constructed with CUSUM analysis showed that all trainees (anesthesiologist residents) included in this study achieved competence (intubation success rates ≥ 80%) in flexible bronchoscope-guided orotracheal intubation. Trainees needed to perform 15 (range, 8 to 25) procedures to achieve proficiency. There was wide variability between trainees.

**Trial registration:**

**Trial registration:** Chinese Clinical Trial Register, ChiCTR 2000032166.

## Introduction

Successful airway management is a key component of patient safety. Endotracheal intubation is an essential skill for anesthesiologists, who must be familiar with various airway management devices and techniques. The flexible bronchoscope is a well-recognized airway management technique that is part of every difficult airway management algorithm and the gold standard for the management of most difficult airways [1–4]. The flexible bronchoscope provides operators “look around the corner” vision and high first-attempt intubation success, and is safe and well tolerated by patients [5–7].

Flexible bronchoscopy is a complex process that requires operator competence to minimize patient risk of complications, such as failed intubation, patient discomfort, hypoxemia, cardiac arrhythmias, myocardial infarction, respiratory depression, cardiorespiratory arrest, severe bleeding, pneumothorax, or mortality. Training in flexible bronchoscopy is multifacilated and must incorporate clinically relevant scenarios. Trainees in flexible bronchoscopy must be familiar with normal airway anatomy and learn technical skills, the indications, contraindications, risks and benefits of the procedure, and communication within a healthcare team and with patients. Skill acquisition and knowledge must reach an appropriate level before trainees perform independent practice on patients. However, there are a paucity of evidence-based outcome measures of trainee competence in performing flexible bronchoscopy.

A cumulative sum (CUSUM) chart is a statistical tool that can model a variable representing performance, productivity or efficiency as a function of another variable reflecting time or experience [8]. CUSUM charts were originally used to monitor processes in manufacturing industries but have recently been applied in healthcare to evaluate learning curves for surgical [9–12] and anesthetic procedures, such as epidural anesthesia, spinal and epidural anesthesia, central venous and arterial cannulation, and orotracheal intubation using direct laryngoscopy [13–17]. CUSUM charts emphasize failures; therefore, trainees with an unsatisfactory performance are quickly identified and immediate corrective measures can be taken.

The objectives of this study were to 1) construct a learning curve for flexible bronchoscope-guided orotracheal intubation for anesthesiology residents using the CUSUM method and 2) determine the number of procedures required to achieve proficiency.

## Methods

### Study design and setting

The study was conducted in Liaocheng People’s Hospital between August 2020 and January 2021. The protocol was approved by the Ethics Committee of Liaocheng People’s Hospital (2020006). The study was registered in the Chinese Clinical Trial Registry (ChiCTR 2000032166). Written informed consent was obtained from residents and patients.

### Participants

Trainee inclusion criteria were: 1) first year anesthesiology resident at Liaocheng People’s Hospital; 2) acquired proficiency in tracheal intubation by direct laryngoscopy and video laryngoscopy; and 3) no previous experience with bronchoscope-guided intubation.

All trainees attended a theoretical lecture on using flexible bronchoscopy, observed a demonstration by an experienced anesthesiologist, and practiced for ≤ 1 hour on a commercially available bronchoscopy simulator (Olympus Company, BF-180). Within 1 week, trainees were permitted to practice on patients.

Patient inclusion criteria were: 1) age ≥ 18 years; 2) American Society of Anesthesiologists (ASA) physical status score Ⅰ~Ⅱ; and 3) undergoing elective surgery under general anesthesia with orotracheal intubation. Patient exclusion criteria were: 1) placement of a double-lumen endotracheal tube; 2) anticipated difficult airway; or 3) awake tracheal intubation.

### Procedures

Patients were positioned supine on the operating table with a thin pillow under their head. Standard monitoring was initiated, including heart rate, electrocardiography, noninvasive blood pressure, oxygen saturation (SpO_2_), and capnography. After pre-oxygenation for 3 minutes, general anesthesia was induced with fentanyl or sufentanil, propofol, and cisatracurium. Mask ventilation with 100% oxygen was provided until complete muscle relaxation. A conventional polyvinylchloride or reinforced tracheal tube (7.5 mm for males, 7.0mm for females) was threaded over a flexible bronchoscope (4.5 mm diameter; TIC-SD-Ш; Zhejiang UE Medical Instrument Co., LTD, Zhejiang, China). Endotracheal intubation was performed by trainees under the supervision of an instructor. The trainee stood above the patient’s head holding the body of the bronchoscope in one hand and the insertion cord in the other. The flexible bronchoscope was inserted into the patient’s oral cavity and passed through the glottis into the trachea, such that the tracheal rings and carina were visible. The tracheal tube was advanced through the glottis under bronchoscopic guidance. The position of the tracheal tube was confirmed visually and by capnography. During intubation, an assistant opened the patient’s mouth and applied the jaw-thrust maneuver to facilitate advancement of the tracheal tube.

If the patient’s SpO_2_ dropped below 90%, intubation was immediately paused, and mask ventilation was used to raise the SpO_2_. Successful intubation was defined as flexible bronchoscope-guided tracheal intubation by trainees. Failed intubation was related to the inability to place the flexible bronchoscope or tracheal tube into the trachea by trainees, including, but not limited to, being unable to advance the bronchoscope through the vocal cords or advance the tracheal tube into the trachea. An intubation attempt was defined as entry of the flexible bronchoscope into the oral cavity. If the first intubation attempt failed, the trainee was permitted one additional attempt. Failure was defined as two unsuccessful attempts, after which the instructor secured the airway. Each trainee’s performance was assessed by the same single observer. Dental, mucosa, arytenoid or vocal cord trauma events associated with intubation were recorded.

Time taken for intubation was defined as the time from entry of the flexible bronchoscope into the oral cavity to withdrawal of the flexible bronchoscope from the oral cavity after successful intubation.

### Data analysis

The CUSUM method was used to evaluate the learning curve for flexible bronchoscope-guided orotracheal intubation for first year anesthesiology residents. CUSUM is a sequential analysis of the cumulative performance of a dichotomized variable.

A CUSUM curve, showing the cumulative sum of deviations from the target value, around zero, for individual measurements was plotted. The x-axis was the timeline and the y axis was the cumulative sum of successes (S) and failures (1-S) of flexible bronchoscope-guided orotracheal intubation. The probability of type Ⅰ (α) and type Ⅱ (β) errors were specified as 0.1. Acceptable and unacceptable failure rates (p_0_ and p_1_, respectively, where p_1_ was set at twice p_0_) were specified as 20% and 40%, as the success rate of flexible bronchoscope-guided orotracheal intubation has been reported at 78–100%. [3,18–20]. The lower decision boundary H_0_ was -2.24 and the upper decision boundary H_1_ was 2.24. The minimum number of procedures needed to assess flexible bronchoscope-guided orotracheal intubation was calculated as 19 (**Table 1**).

**Table 1:** Parameters and calculations used for the CUSUM analysis.

Parameters	Explanation / calculation	Numerical values
α	Probability of type Ⅰ error	0.1
β	Probability of type Ⅱ error	0.1
p_0_	Acceptable failure rate	0.20
p_1_	Unacceptable failure rate	0.40
a	Ln [(1-β) / α]	2.197
b	Ln [(1-α) /β]	2.197
P	Ln (P_0_ / P_1_)	0.693
Q	Ln [(1- p_0_) / (1- p_1_)]	0.288
S	Q / (P+Q)	0.293
H_0_	-b / (P+Q)	-2.24
H_1_	a / (P+Q)	2.24
N_0_	Sample size with p_0_ [H_0_ (1-α)- αH_1_] / (S-p_0_)	19
N_1_	Sample size with p_1_ [H_1_(1-β)–βH_0_] / (p_1_-S)	17

If performance was a failure, the CUSUM sloped upward. If the CUSUM line rose above the upper decision boundary (H_1_), the trainee was considered incompetent for flexible bronchoscope-guided orotracheal intubation. If performance was successful, the CUSUM sloped downward. If the CUSUM line dropped below the lower decision boundary (H_0_), the trainee was considered competent for flexible bronchoscope-guided orotracheal intubation. If the CUSUM line remained between H_0_ and H_1_, training should have continued.

## Results

This study included 12 trainees (first year anesthesiology residents). All trainees practiced on the bronchoscopy simulator for ≤ 1 hour. Trainees performed flexible bronchoscope-guided orotracheal intubation on 364 patients.

First-attempt intubation success occurred in 317 (87.1%) patients. Second-attempt intubation success occurred in 23 (6.3%) patients. Overall, the flexible bronchoscope-guided orotracheal intubation success rate was 93.4% (range, 85.3% to 100%). Instructors secured the airways in 24 (6.6%) patients.

**Fig 1:**
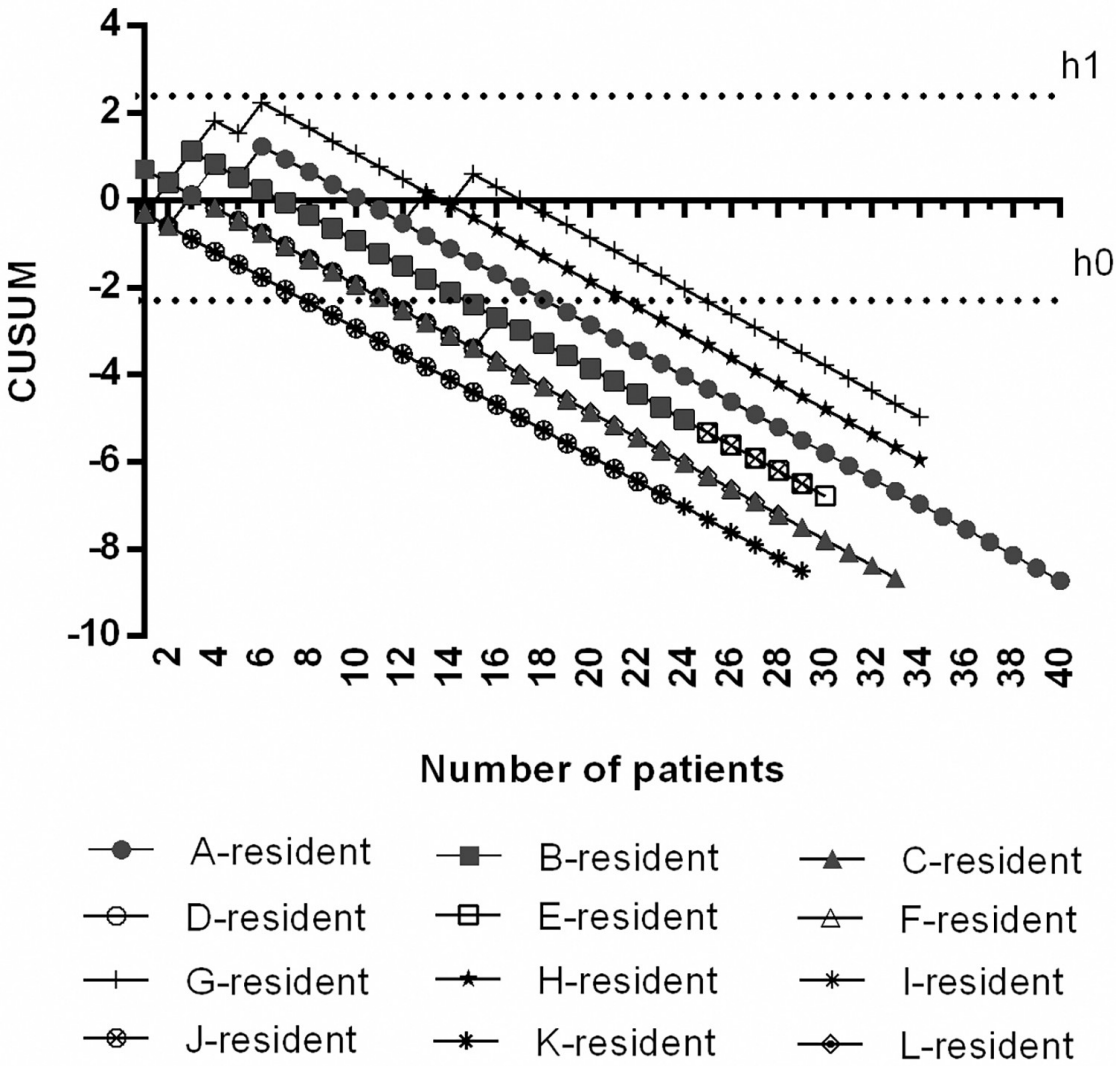
CUSUM curves for bronchoscope-guided orotracheal intubation. Lines A—L represent the learning curves for each trainee (anesthesiologist residents). The lower decision boundary H_0_ was -2.24 and the upper decision boundary H_1_ was 2.24.

The mean number of orotracheal intubation procedures per trainee was 31 ± 5 (range, 23 to 40). All trainees crossed the lower decision boundary (H_0_) after 15.1 ± 5.6 procedures (range, 8 to 25 procedures). The individual performance of each trainee is shown in **Table 2**. CUSUM curves for each trainee are shown in **Fig 1**.

**Table 2:** Individual performance of each trainee.

Resident	Patients	Successes	Success rate (%)	No. patients needed to cross H_0_
A	40	37	92.5	22
B	24	22	91.7	15
C	33	32	97.0	12
D	23	23	100.0	8
E	30	28	93.3	15
F	31	30	96.8	12
G	34	29	85.3	25
H	34	30	88.2	22
I	29	26	89.7	18
J	29	27	93.1	12
K	29	29	100.0	8
L	28	27	96.4	12
Total	364	340	93.67±4.57	15.08±5.57

**Fig 2:**
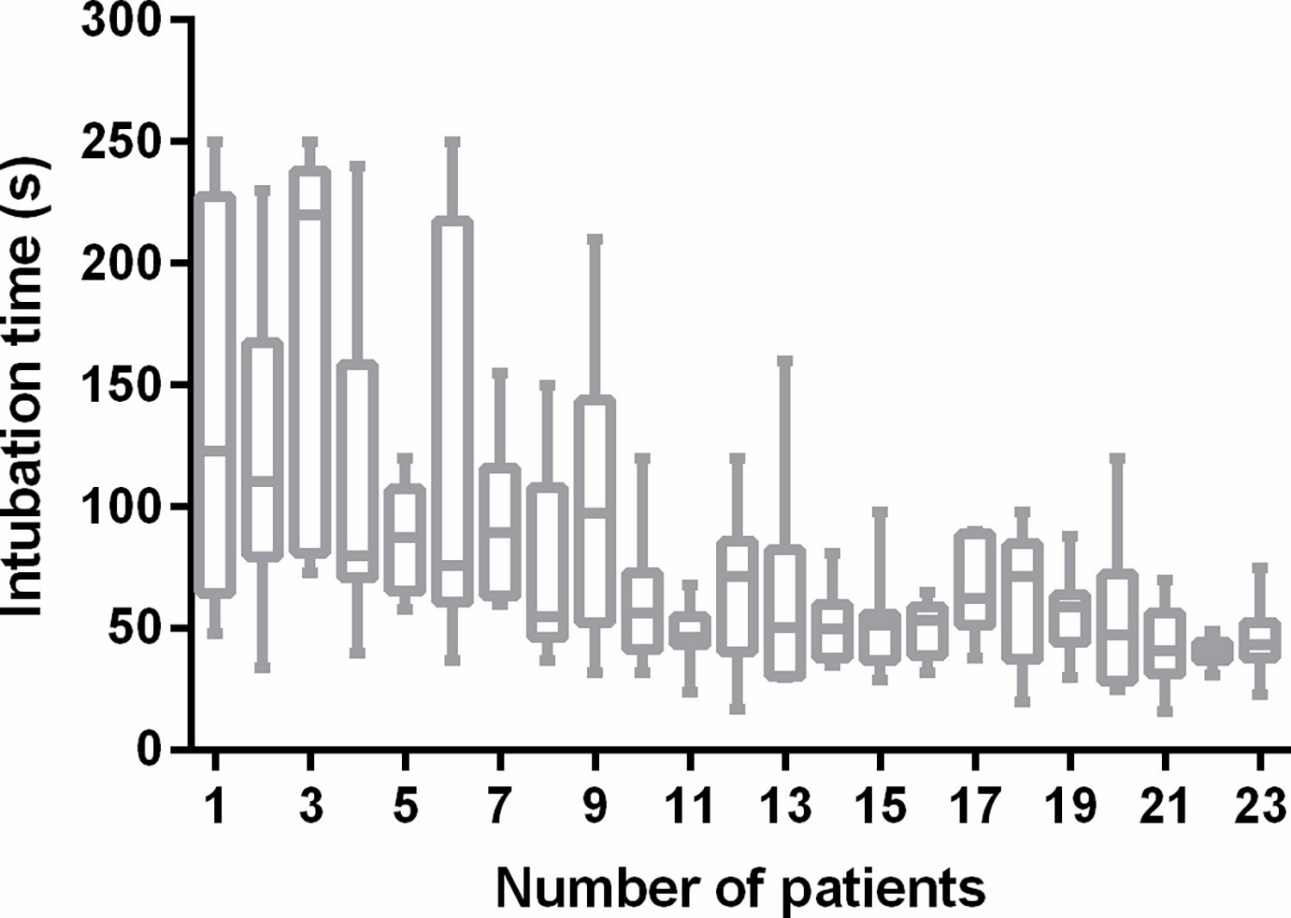
Time for orotracheal intubation. Data are median (interquartile range) with min and max.

The minimum and maximum time for orotracheal intubation was 16 and 250 seconds, respectively. Time to intubation was stable after performing the procedure in 9 patients (**Fig 2**).

There were significant increases in first-attempt intubation success rate [77.5% (IQR: 72.3%, 88.6%) vs. 100% (IQR: 94.5%, 100%)] and overall intubation success rate [88.3% (IQR: 83.1%, 90.9%) vs. 100% (IQR: 100%, 100%)] and a significant decrease in median intubation time [39s (IQR: 30, 50) vs. 76s (IQR: 54, 119)] (all *P* < 0.001, **Table 3**) after crossing the lower decision boundary (H_0_) compared to before crossing the lower decision boundary (H_0_).

**Table 3:** Intubation success rate and time before and after crossing the lower decision boundary (H_0_).

	Before crossing H_0_, median (IQR)	After crossing H_0_, median (IQR)	*P* value
First-attempt intubation success rate (%)	77.5 (72.3, 88.6)	100 (94.5, 100)	0.001
Overall intubation success rate (%)	88.3 (83.1, 90.9)	100 (100, 100)	<0.001
Intubation time (s)	76 (54, 119)	39 (30, 50)	<0.001

There were no dental, mucosa, arytenoid or vocal cord trauma events associated with intubation.

## Discussion

The objective of this study was to construct a learning curve for flexible bronchoscope-guided orotracheal intubation for anesthesiology residents using the CUSUM method and determine the number of procedures required to achieve proficiency. Findings showed that anesthesiology residents with no previous experience with bronchoscope-guided intubation had to perform a mean of 15 procedures in patients with normal airways under general anesthesia to achieve a flexible bronchoscope-guided orotracheal intubation success rate of 80%. There was a wide variability in the number of procedures each trainee needed to perform to achieve proficiency, indicating that trainees learned at different speeds.

Flexible bronchoscope-guided intubation can be performed nasally or orally in awake patients with topical or regional anesthesia alone, or in sedated or anesthetized patients. The nasal route is often used on patients with poor mouth opening or a large tongue as it courses past the nasopharynx with less obstruction by the tongue [21]. Awake flexible bronchoscope-guided intubation is strongly recommended in patients with anticipated difficult airways [2,6,22,23]. In this study, orotracheal intubation was performed in anesthetized apneic patients with normal airways as it is considered more difficult than nasal intubation of the awake patient. In anesthetized patients, relaxation of oral and pharyngeal muscles allows the larynx to become more anterior relative to other structures. Trainees were expected to be able to use the intubation techniques learned in anesthetized patients in the awake patient with minimal modification.

A previous study showed anesthesiologists achieved competence (first-attempt intubation success rates > 90% and intubation time of ≤ 2 minutes) in fiberoptic laryngoscopy and intubation in anesthetized patients with normal airways after 10 procedures [22], and all the trainees learned at the same rate. Several reasons may explain the disparate findings between the prior report and the present study. First, the prior report used logarithmic analysis of the mean (+/- SD) time for intubation of patients for all residents combined to generate learning curves, while the present study used the CUSUM method. Second, there was differential use of airway devices and techniques, such as the oropharyngeal airway, bite block, tongue root holder, and jaw thrust maneuver [24,25] that can facilitate airway opening and improve the intubation success rate. The prior report used a bite block to prevent airway obstruction and keep the flexible bronchoscope centered, thus facilitating intubation. In the present study, the jaw thrust was used as a positioning maneuver. In 6 (26.1%) patients with a failed first-attempt intubation, second-attempt intubation was successful after the jaw thrust maneuver was modified. Third, each study used a different type of flexible bronchoscope, which may explain differences in first-attempt intubation success rates (prior report: 93.5% vs. present study: 87.1%).

In the present study, patient factors, including the presence of secretions that impaired visibility, caused difficulties during flexible bronchoscope-guided orotracheal intubation in 6 (26.1%) of the 23 patients who underwent two endotracheal intubation attempts [26]. No patient received antisialagogues before surgery. Other challenges when performing this procedure include difficulties advancing the tracheal tube due to differences in the inner diameter of the endotracheal tube and the outer diameter of the flexible bronchoscope [19].

This study was associated with several limitations. First, only 12 residents in a single center were evaluated, and inter-individual differences were large. The minimum number of procedures required to master flexible bronchoscope-guided orotracheal intubation may not be applicable to more novice learners. Second, only adult patients with normal airways were included, and it is not clear if trainees can transfer their skills to obese patients or those with difficult airways, Third, trainees practiced on the bronchoscopy simulator for ≤ 1 hour. If simulation practice was increased, the number of procedures needed to achieve proficiency in flexible bronchoscope-guided orotracheal intubation may be decreased. Finally, a scoring system was not used to assess the performance of the trainees.

## Conclusion

Learning curves constructed with CUSUM analysis showed that all trainees (anesthesiologist residents) included in this study achieved competence (intubation success rates ≥ 80%) in flexible bronchoscope-guided orotracheal intubation. Trainees needed to perform 15 (range, 8 to 25) procedures to achieve proficiency. There was wide variability between trainees.

## Supporting information

S1 File (DOCX)
