# Supplementary material for: Learning curve for flexible bronchoscope-guided orotracheal intubation for anesthesiology residents: A cumulative sum analysis
Source: PLoS One. 2023 Jul 13;18(7):e0288617. doi: 10.1371/journal.pone.0288617 (PMC10343144; doi:10.1371/journal.pone.0288617)
Supplement: S1 File — (DOCX) [file pone.0288617.s001.docx]

**Supporting information**

**S1 Data**

| Resident | Patient | First-attempt intubation success (1=success, 2=no success) | Second-attempt intubation success (1=success, 2=no success,-=NA) | Intubation time |
| --- | --- | --- | --- | --- |
| A | 1 | 2 | 2 | 250 |
| A | 2 | 1 | - | 94 |
| A | 3 | 2 | 1 | 120 |
| A | 4 | 2 | 2 | 240 |
| A | 5 | 2 | 1 | 90 |
| A | 6 | 2 | 2 | 250 |
| A | 7 | 1 | - | 67 |
| A | 8 | 1 | - | 52 |
| A | 9 | 1 | - | 98 |
| A | 10 | 1 | - | 40 |
| A | 11 | 1 | - | 45 |
| A | 12 | 1 | - | 120 |
| A | 13 | 1 | - | 30 |
| A | 14 | 1 | - | 50 |
| A | 15 | 2 | 1 | 56 |
| A | 16 | 1 | - | 60 |
| A | 17 | 1 | - | 52 |
| A | 18 | 1 | - | 72 |
| A | 19 | 1 | - | 87 |
| A | 20 | 1 | - | 25 |
| A | 21 | 1 | - | 31 |
| A | 22 | 1 | - | 48 |
| A | 23 | 1 | - | 23 |
| A | 24 | 1 | - | 24 |
| A | 25 | 1 | - | 25 |
| A | 26 | 1 | - | 28 |
| A | 27 | 1 | - | 29 |
| A | 28 | 1 | - | 24 |
| A | 29 | 1 | - | 25 |
| A | 30 | 1 | - | 26 |
| A | 31 | 1 | - | 22 |
| A | 32 | 1 | - | 30 |
| A | 33 | 1 | - | 28 |
| A | 34 | 1 | - | 32 |
| A | 35 | 2 | 1 | 34 |
| A | 36 | 1 | - | 30 |
| A | 37 | 1 | - | 29 |
| A | 38 | 1 | - | 28 |
| A | 39 | 1 | - | 32 |
| A | 40 | 1 | - | 35 |
| B | 1 | 2 | 2 | 220 |
| B | 2 | 1 | - | 130 |
| B | 3 | 2 | 2 | 230 |
| B | 4 | 1 | - | 80 |
| B | 5 | 2 | 1 | 65 |
| B | 6 | 1 | - | 60 |
| B | 7 | 1 | - | 89 |
| B | 8 | 1 | - | 110 |
| B | 9 | 1 | - | 158 |
| B | 10 | 1 | - | 55 |
| B | 11 | 1 | - | 43 |
| B | 12 | 1 | - | 85 |
| B | 13 | 1 | - | 84 |
| B | 14 | 1 | - | 80 |
| B | 15 | 1 | - | 56 |
| B | 16 | 1 | - | 53 |
| B | 17 | 1 | - | 69 |
| B | 18 | 1 | - | 77 |
| B | 19 | 1 | - | 63 |
| B | 20 | 1 | - | 120 |
| B | 21 | 1 | - | 45 |
| B | 22 | 1 | - | 41 |
| B | 23 | 1 | - | 75 |
| B | 24 | 1 | - | 36 |
| C | 1 | 2 | 1 | 123 |
| C | 2 | 1 | - | 180 |
| C | 3 | 2 | 2 | 250 |
| C | 4 | 1 | - | 170 |
| C | 5 | 1 | - | 109 |
| C | 6 | 1 | - | 210 |
| C | 7 | 1 | - | 126 |
| C | 8 | 1 | - | 150 |
| C | 9 | 1 | - | 145 |
| C | 10 | 1 | - | 74 |
| C | 11 | 1 | - | 49 |
| C | 12 | 1 | - | 75 |
| C | 13 | 1 | - | 95 |
| C | 14 | 1 | - | 38 |
| C | 15 | 1 | - | 41 |
| C | 16 | 1 | - | 39 |
| C | 17 | 1 | - | 89 |
| C | 18 | 1 | - | 87 |
| C | 19 | 1 | - | 59 |
| C | 20 | 1 | - | 77 |
| C | 21 | 1 | - | 60 |
| C | 22 | 1 | - | 31 |
| C | 23 | 1 | - | 38 |
| C | 24 | 1 | - | 45 |
| C | 25 | 1 | - | 45 |
| C | 26 | 1 | - | 39 |
| C | 27 | 1 | - | 42 |
| C | 28 | 1 | - | 40 |
| C | 29 | 1 | - | 32 |
| C | 30 | 1 | - | 38 |
| C | 31 | 1 | - | 30 |
| C | 32 | 1 | - | 36 |
| C | 33 | 1 | - | 32 |
| D | 1 | 1 | - | 48 |
| D | 2 | 1 | - | 34 |
| D | 3 | 2 | 1 | 73 |
| D | 4 | 1 | - | 68 |
| D | 5 | 2 | 1 | 120 |
| D | 6 | 1 | - | 70 |
| D | 7 | 1 | - | 155 |
| D | 8 | 1 | - | 41 |
| D | 9 | 1 | - | 210 |
| D | 10 | 1 | - | 57 |
| D | 11 | 1 | - | 48 |
| D | 12 | 1 | - | 36 |
| D | 13 | 2 | 1 | 38 |
| D | 14 | 1 | - | 43 |
| D | 15 | 1 | - | 29 |
| D | 16 | 1 | - | 65 |
| D | 17 | 1 | - | 90 |
| D | 18 | 1 | - | 37 |
| D | 19 | 1 | - | 45 |
| D | 20 | 2 | 1 | 46 |
| D | 21 | 2 | 1 | 34 |
| D | 22 | 1 | - | 40 |
| D | 23 | 1 | - | 75 |
| E | 1 | 2 | 1 | 62 |
| E | 2 | 2 | 2 | 220 |
| E | 3 | 2 | 2 | 240 |
| E | 4 | 1 | - | 124 |
| E | 5 | 1 | - | 85 |
| E | 6 | 1 | - | 63 |
| E | 7 | 2 | 1 | 85 |
| E | 8 | 1 | - | 112 |
| E | 9 | 1 | - | 140 |
| E | 10 | 2 | 1 | 120 |
| E | 11 | 1 | - | 68 |
| E | 12 | 1 | - | 80 |
| E | 13 | 1 | - | 78 |
| E | 14 | 1 | - | 60 |
| E | 15 | 1 | - | 53 |
| E | 16 | 1 | - | 56 |
| E | 17 | 1 | - | 88 |
| E | 18 | 2 | 1 | 98 |
| E | 19 | 1 | - | 60 |
| E | 20 | 1 | - | 54 |
| E | 21 | 2 | 1 | 70 |
| E | 22 | 1 | - | 43 |
| E | 23 | 1 | - | 48 |
| E | 24 | 1 | - | 64 |
| E | 25 | 1 | - | 89 |
| E | 26 | 1 | - | 36 |
| E | 27 | 1 | - | 44 |
| E | 28 | 1 | - | 45 |
| E | 29 | 1 | - | 32 |
| E | 30 | 1 | - | 38 |
| F | 1 | 1 | - | 60 |
| F | 2 | 1 | - | 84 |
| F | 3 | 2 | 2 | 230 |
| F | 4 | 1 | - | 40 |
| F | 5 | 2 | 1 | 66 |
| F | 6 | 1 | - | 37 |
| F | 7 | 1 | - | 60 |
| F | 8 | 1 | - | 50 |
| F | 9 | 1 | - | 32 |
| F | 10 | 1 | - | 32 |
| F | 11 | 1 | - | 24 |
| F | 12 | 1 | - | 17 |
| F | 13 | 1 | - | 30 |
| F | 14 | 1 | - | 35 |
| F | 15 | 1 | - | 35 |
| F | 16 | 1 | - | 32 |
| F | 17 | 1 | - | 38 |
| F | 18 | 1 | - | 20 |
| F | 19 | 1 | - | 30 |
| F | 20 | 1 | - | 30 |
| F | 21 | 1 | - | 16 |
| F | 22 | 1 | - | 45 |
| F | 23 | 1 | - | 45 |
| F | 24 | 1 | - | 30 |
| F | 25 | 1 | - | 25 |
| F | 26 | 1 | - | 32 |
| F | 27 | 1 | - | 28 |
| F | 28 | 1 | - | 33 |
| F | 29 | 1 | - | 35 |
| F | 30 | 1 | - | 30 |
| F | 31 | 1 | - | 29 |
| G | 1 | 2 | 2 | 250 |
| G | 2 | 1 | - | 96 |
| G | 3 | 2 | 2 | 250 |
| G | 4 | 2 | 2 | 240 |
| G | 5 | 1 | - | 91 |
| G | 6 | 2 | 2 | 250 |
| G | 7 | 1 | - | 110 |
| G | 8 | 1 | - | 52 |
| G | 9 | 1 | - | 97 |
| G | 10 | 1 | - | 39 |
| G | 11 | 2 | 1 | 44 |
| G | 12 | 1 | - | 118 |
| G | 13 | 1 | - | 31 |
| G | 14 | 1 | - | 52 |
| G | 15 | 2 | 2 | 55 |
| G | 16 | 1 | - | 62 |
| G | 17 | 1 | - | 51 |
| G | 18 | 1 | - | 71 |
| G | 19 | 1 | - | 88 |
| G | 20 | 1 | - | 26 |
| G | 21 | 1 | - | 32 |
| G | 22 | 1 | - | 49 |
| G | 23 | 1 | - | 23 |
| G | 24 | 1 | - | 44 |
| G | 25 | 1 | - | 45 |
| G | 26 | 1 | - | 38 |
| G | 27 | 1 | - | 42 |
| G | 28 | 1 | - | 41 |
| G | 29 | 1 | - | 34 |
| G | 30 | 1 | - | 39 |
| G | 31 | 1 | - | 32 |
| G | 32 | 1 | - | 37 |
| G | 33 | 1 | - | 42 |
| G | 34 | 1 | - | 40 |
| H | 1 | 2 | 2 | 230 |
| H | 2 | 1 | - | 130 |
| H | 3 | 2 | 2 | 232 |
| H | 4 | 1 | - | 80 |
| H | 5 | 1 | - | 65 |
| H | 6 | 2 | 2 | 220 |
| H | 7 | 1 | - | 90 |
| H | 8 | 1 | - | 37 |
| H | 9 | 2 | 1 | 42 |
| H | 10 | 1 | - | 56 |
| H | 11 | 1 | - | 44 |
| H | 12 | 1 | - | 86 |
| H | 13 | 2 | 2 | 160 |
| H | 14 | 1 | - | 81 |
| H | 15 | 1 | - | 57 |
| H | 16 | 1 | - | 54 |
| H | 17 | 1 | - | 70 |
| H | 18 | 1 | - | 78 |
| H | 19 | 1 | - | 64 |
| H | 20 | 1 | - | 58 |
| H | 21 | 1 | - | 44 |
| H | 22 | 1 | - | 40 |
| H | 23 | 1 | - | 54 |
| H | 24 | 1 | - | 25 |
| H | 25 | 1 | - | 26 |
| H | 26 | 1 | - | 29 |
| H | 27 | 1 | - | 30 |
| H | 28 | 1 | - | 25 |
| H | 29 | 1 | - | 26 |
| H | 30 | 1 | - | 27 |
| H | 31 | 1 | - | 22 |
| H | 32 | 1 | - | 31 |
| H | 33 | 1 | - | 29 |
| H | 34 | 1 | - | 32 |
| I | 1 | 2 | 1 | 123 |
| I | 2 | 2 | 2 | 230 |
| I | 3 | 2 | 2 | 210 |
| I | 4 | 1 | - | 92 |
| I | 5 | 1 | - | 120 |
| I | 6 | 2 | 2 | 210 |
| I | 7 | 1 | - | 118 |
| I | 8 | 1 | - | 102 |
| I | 9 | 1 | - | 136 |
| I | 10 | 1 | - | 78 |
| I | 11 | 1 | - | 56 |
| I | 12 | 1 | - | 68 |
| I | 13 | 1 | - | 65 |
| I | 14 | 1 | - | 50 |
| I | 15 | 1 | - | 50 |
| I | 16 | 1 | - | 39 |
| I | 17 | 1 | - | 89 |
| I | 18 | 1 | - | 87 |
| I | 19 | 1 | - | 59 |
| I | 20 | 1 | - | 77 |
| I | 21 | 1 | - | 60 |
| I | 22 | 1 | - | 31 |
| I | 23 | 1 | - | 38 |
| I | 24 | 1 | - | 34 |
| I | 25 | 1 | - | 29 |
| I | 26 | 1 | - | 32 |
| I | 27 | 1 | - | 33 |
| I | 28 | 1 | - | 29 |
| I | 29 | 1 | - | 30 |
| J | 1 | 2 | 2 | 186 |
| J | 2 | 1 | - | 125 |
| J | 3 | 1 | - | 78 |
| J | 4 | 1 | - | 69 |
| J | 5 | 1 | - | 102 |
| J | 6 | 1 | - | 82 |
| J | 7 | 1 | - | 98 |
| J | 8 | 1 | - | 45 |
| J | 9 | 1 | - | 50 |
| J | 10 | 1 | - | 65 |
| J | 11 | 1 | - | 52 |
| J | 12 | 1 | - | 54 |
| J | 13 | 1 | - | 49 |
| J | 14 | 1 | - | 43 |
| J | 15 | 1 | - | 40 |
| J | 16 | 2 | 2 | 50 |
| J | 17 | 1 | - | 56 |
| J | 18 | 1 | - | 38 |
| J | 19 | 1 | - | 44 |
| J | 20 | 1 | - | 42 |
| J | 21 | 1 | - | 38 |
| J | 22 | 1 | - | 40 |
| J | 23 | 1 | - | 42 |
| J | 24 | 1 | - | 39 |
| J | 25 | 1 | - | 74 |
| J | 26 | 1 | - | 43 |
| J | 27 | 1 | - | 51 |
| J | 28 | 1 | - | 48 |
| J | 29 | 1 | - | 36 |
| K | 1 | 1 | - | 86 |
| K | 2 | 1 | - | 78 |
| K | 3 | 1 | - | 80 |
| K | 4 | 1 | - | 76 |
| K | 5 | 1 | - | 82 |
| K | 6 | 1 | - | 66 |
| K | 7 | 1 | - | 62 |
| K | 8 | 1 | - | 88 |
| K | 9 | 1 | - | 80 |
| K | 10 | 1 | - | 70 |
| K | 11 | 1 | - | 66 |
| K | 12 | 1 | - | 65 |
| K | 13 | 1 | - | 52 |
| K | 14 | 1 | - | 39 |
| K | 15 | 2 | 1 | 98 |
| K | 16 | 1 | - | 56 |
| K | 17 | 1 | - | 52 |
| K | 18 | 1 | - | 48 |
| K | 19 | 1 | - | 50 |
| K | 20 | 1 | - | 49 |
| K | 21 | 1 | - | 46 |
| K | 22 | 1 | - | 43 |
| K | 23 | 1 | - | 45 |
| K | 24 | 1 | - | 52 |
| K | 25 | 1 | - | 50 |
| K | 26 | 1 | - | 35 |
| K | 27 | 1 | - | 44 |
| K | 28 | 1 | - | 45 |
| K | 29 | 1 | - | 32 |
| L | 1 | 1 | - | 72 |
| L | 2 | 2 | 2 | 66 |
| L | 3 | 1 | - | 84 |
| L | 4 | 1 | - | 76 |
| L | 5 | 1 | - | 58 |
| L | 6 | 1 | - | 59 |
| L | 7 | 1 | - | 60 |
| L | 8 | 1 | - | 58 |
| L | 9 | 1 | - | 60 |
| L | 10 | 1 | - | 45 |
| L | 11 | 1 | - | 34 |
| L | 12 | 1 | - | 27 |
| L | 13 | 1 | - | 30 |
| L | 14 | 1 | - | 38 |
| L | 15 | 1 | - | 36 |
| L | 16 | 1 | - | 35 |
| L | 17 | 1 | - | 40 |
| L | 18 | 1 | - | 31 |
| L | 19 | 1 | - | 30 |
| L | 20 | 1 | - | 28 |
| L | 21 | 1 | - | 25 |
| L | 22 | 1 | - | 36 |
| L | 23 | 1 | - | 38 |
| L | 24 | 1 | - | 29 |
| L | 25 | 1 | - | 26 |
| L | 26 | 1 | - | 25 |
| L | 27 | 1 | - | 27 |
| L | 28 | 1 | - | 26 |
